# Estimating the risk of SARS-CoV-2 deaths using a Markov switching-volatility model combined with heavy-tailed distributions for South Africa

**DOI:** 10.1186/s12889-022-14249-8

**Published:** 2022-10-07

**Authors:** Nobuhle Mthethwa, Retius Chifurira, Knowledge Chinhamu

**Affiliations:** School of Mathematics, Statistics and Computer Science, University of KwaZulu- Natal, Durban, South Africa

**Keywords:** SARS-CoV-2 (Covid-19) infection, Death, Volatility models, Value-at-risk, Heavy-tailed distributions, Structural breaks

## Abstract

**Background:**

SARS-CoV-2 (Covid-19 virus) infection exposed the unpreparedness of African countries to health-related issues, South Africa included. Africa recorded more than 211 853 deaths as a consequence of Covid-19. When rare and deadly diseases require urgent hospitalisation strikes, governments and healthcare providers are usually caught unprepared, resulting in huge loss of lives. Usually, at the beginning of such pandemics, there is no rich data for health practitioners and academics to be able to forecast the number of patients or deaths related to the pandemic. This study aims to predict the number of deaths associated with Covid-19 infection. With the availability of the number of deaths on a daily basis, the results stemming from this study are important to inform and plan health policy.

**Methods:**

This study uses the daily number of deaths due to Covid-19 infection. Exploratory data analysis reveals that the data exhibits non-normality, three structural breaks and volatility clustering characteristics. The Markov switching (MS)-generalized autoregressive conditional heteroscedasticity (GARCH)-type model combined with heavy-tailed distributions is fitted to the returns of the data. Using available daily reported Covid-19-related deaths up until 26 August 2021, we report 10-day ahead forecasts of deaths. All forecasts are compared to the actual observed values in the forecasting period.

**Results:**

The Anderson–Darling Goodness of fit test confirms that the fitted models are adequate for the data. The Kupiec likelihood ratio test and the root mean square error (RMSE) were used to select the robust model at different risk levels. At 95% the MS(3)-GARCH(1,1) combined with Pearson’s type IV distribution (PIVD) is the best model. This indicates that the proposed best-fitting model is reasonable and can be used for predicting the daily number of deaths due to Covid-19.

**Conclusion:**

The MS(3)-GARCH(1,1)-PIVD model provides a reliable and accurate method for predicting the minimum number of death due to Covid-19. The accuracy of the proposed model will assist policymakers, academics and health practitioners in forecasting the volatility of future health-related deaths in which the predictability of volatility plays an integral role in health risk management.

## Introduction

SARS-CoV-2 a virus is commonly known as the Covid-19 virus, first emerged in Wuhan, China, in December 2019 [1]. The virus rapidly spread to over 100 countries; South Africa included. Africa is a large population with a compromised immune system, has a high prevalence of diseases like Human Immunodeficiency Virus/Acquired Immunodeficiency syndrome, Malaria, Tuberculosis and many more [2]. The continent has a weak health care system and a poor economic discipline, and for these reasons, Africa is different from the other continents that are presently dealing with Covid-19 [2].

In South Africa, the first case of covid-19 was reported on the 5^th^ of March 2020, which was a result of international travel from Italy which led to the transmission of the virus locally [3]. Numerous ways of curbing the spread of Covid-19 were communicated, including social distancing, regular sanitizing and washing of hands, quarantine, and lockdown [4]. Lockdown restricts people from leaving or entering buildings or other locations; this is done as a security measure in emergencies. The implementation of lockdown resulted in the closing of borders, the closure of schools and later the implementation of online learning in schools, and the closure of businesses. Further restrictions were imposed which included the banning of alcohol, closing of clubs and entertainment areas, the closing of churches, anything and everything that involved public gatherings. Even in funerals, a certain number of people was prohibited. Businesses reported a significant decline in their returns because of lockdown restrictions, especially those classified as "non-essential” [5]. Besides the threats posed by Covid-19, South Africa had pre-existing issues, including unstable economic growth, high unemployment rate, falling per capita income and unviable government debt trends [6].

South Africa was hit the hardest by Covid-19 compared to other countries in Africa, reporting over 2,9 million confirmed cases and over 89 500 Covid-19 related fatalities as of the 19^th^ November 2021. South Africa experienced significant peaks in the number of deaths during the first and second wave of the Covid-19 pandemic, within the 2021 Mid-year Population Estimates (MYPE) period between July 2020 and June 2021. This yielded a noticeable increase in the crude death rate (CDR) from 8,7 deaths per 1 000 people in 2020 to 11,6 deaths per 1 000 people in 2021. The rise in deaths in 2021 (approximated to be 34%), caused the 2021 Life Expectancy (LE) at birth to plunge for South Africa [5].

Shim et al. (2021) [7] estimated the risk of Covid-19 deaths during the outbreak in Korea using time-delay adjusted crude case fatality risk (CFR). The data set used is from the Korea Centers for Disease Control and Prevention (KCDC). The data set is made up of geographic areas: Daegu, Gyeongsanguk-do and other regions and Korea (national). The authors discovered that the extremely affected areas were Gyeongsanguk-do and Daegu, other regions and the rest of Korea have a less severe profile. Furthermore, it was discovered that the fatality risk due to Covid-19 increases with increasing age meaning that the older group exhibits a higher case fatality.

A similar study to the one done by Shim et al*.* [7] was done by Mizumoto and Chowell (2020) [8] in China, where they estimated the risk of death from Covid-19 using the CFR. The data set used is from Hubei province and the city of Wuhan in China. The gamma, exponential and log-normal distributions were used. The results showed that the gamma distribution was the best fit for the delays from hospitalization to death and the log-normal produced the best fit for the delays from illness onset to death.

Nyabadza et al*.* (2020) [4] modelled the impacts of social distancing on Covid-19 for South Africa using the susceptible exposed-infected-removed (SEIR) model. The data was obtained from Coronavirus COVID-19 (2019-nCoV) data repository for South Africa, for March 2020. The model is fitted to the cumulative cases before lockdown and during the lockdown. The model showed that with the implementation of social distancing under the initial lockdown level between March 26 and April 13, 2020, there would be an increase in the number of infected cases. The model also looked at the effect of relaxing the social distancing measures after the first announcement of the lockdown. The results reveal that the relaxation of social distancing would increase the cases by a certain percentage and the opposite is true. The model results correctly forecasted the number of cases after the initial lockdown level was relaxed approaching the end of April 2020. These results have implications for the management and policy direction in the early phase of the epidemic.

Rivera et al. (2020) [9] investigated the excess mortality during Covid-19 in the United States. This study looked at deaths directly and indirectly caused by Covid-19 for 13 states with high Covid-19 deaths, these states are Illinois, New Jersey, Massachusetts, Connecticut, New York, Washington, Colorado, Michigan, California, Florida, Indiana, Pennsylvania and Louisiana. The data used was collected from the National Center for Health Statistics (NCHS) Mortality Surveillance System (MSS) data release. A semiparametric model and a conventional model are used. It was found that the semiparametric model presents more advantages than the conventional approach. The authors concluded that all the states have an excess all-cause that exceeds the number of deaths for Covid-19.

Reddy et al. (2021) [10] and Surowiec and Warowny (2021) predicted the number of deaths due to Covid-19. Reddy et al. (2021) [10] used a data-driven approach to predict the short-term real-time total number of Covid-19 cases and deaths using linear growth curves. Reddy et al. [10] found that linear growth curves provide reliable and accurate forecasts for a maximum period of 10 days ahead. For data that exhibit high volatility, researchers use volatility methods to estimate future returns. The daily number of deaths due to Covid-19 exhibits high volatility clustering behaviour (just like volatility returns). This can be a justification for exploring the use of volatility models in the prediction of the number of deaths due to Covid-19. Volatility models are commonly used to estimate the risk of financial returns [11]. Research on the robustness of volatility models combined with heavy-tailed distributions in estimating financial risk has extensively been done [12–14]. Surowiec and Warowny (2021) [14] employed the Value-at-Risk (VaR) concept to estimate the death rate from Covid-19 infection. Four Central European countries namely, Poland, Hungary, Czech Republic and Slovakia are used as “portfolios”. The data used is from 11^th^ of January 2021 to the 28^th^ of March 2021. The calculation methods report the VaR for the total number of deaths for the four countries over 14 days, the number of deaths is known for the initial 13 days and the 14^th^ day is forecasted. They employ the variance–covariance approach which is a parametric method that assumes that the portfolio components are normal, making the log-returns of the daily deaths normally distributed. According to Cont, (2001) [15] the log-returns have heavier tails compared to the normal distribution and they exhibit volatility clustering.

Therefore, this study departs from the study of Surowiec and Warowny (2021) [14] and Reddy et al. (2021) [10] by incorporating volatility clustering, structural breaks, and heavy-tailed distributions using the Markov-switching GARCH-type models combined with the heavy-tailed distribution in estimating the minimum daily number of deaths due to Covid-19. To the best of our knowledge, there is restricted use of VaR models incorporating the MS-GARCH-type model and heavy-tailed distributions on health-related data. In the literature, we could not find the application of the MS-GARCH-type model combined with heavy-tailed distributions in estimating the risk of deaths due to Covid-19 infection.

This study is interested in estimating the minimum daily number of death due to Covid-19. To approximate the epidemiological and economic burden of infectious disease, it is critical to estimate the minimum number of daily deaths. The methods famously known to be used in Finance are used in this study, as the daily deaths have exhibited similar characteristics as financial returns, such as volatility clustering. Moreover, they exhibit heavier tails than the normal distribution [15].

## Exploratory data analysis and data description

### Data description

The data used in this study is the daily deaths due to COVID-19 for South Africa from the COVID-19 data repository for South Africa, which was constructed, sustained, and hosted by Data Science for social impact research group led by Dr. Vukosi Marivate at the University of Pretoria. The data is collected from the National Institute for Communicable Diseases (NICD) and the Department of Health (DoH). It contains 514 observations from 23 March 2020 to 26 August 2021. The data can be accessed on the website: https://github.com/dsfsi/covid19za/blob/master/data/

### Data exploration

**Fig. 1:**
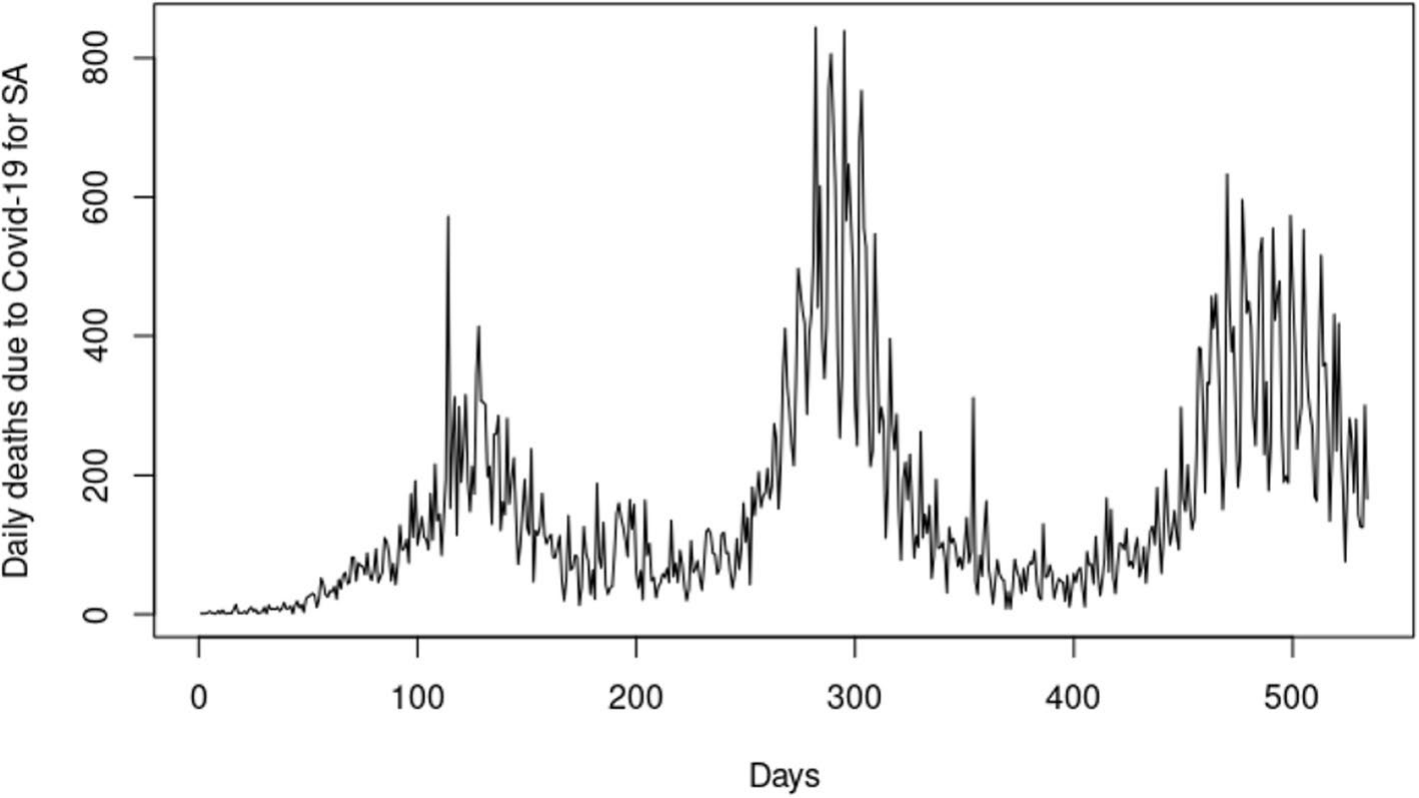
Time series plot of the daily deaths for Covid-19

Figure 1 shows the time series plot for the daily deaths for South Africa. Figure 1 helps understand the trend of the deaths due to Covid-19 in South Africa for 0 to 514 days. Day 0 corresponds to the first record of death on 23 March 2020, and day 514 is the last record of death on 26 August 2021.

In Fig. 1, the number of daily deaths indicates an increasing/upward trend, resulting in the data being non-stationary. There is also some highs and lows (bumps), which could be due to potential structural breaks in the data. Table 1 displays the tests for stationarity using the augmented Dickey-Fuller (ADF) and Phillips-Perron (PP) tests.

**Table 1:** *P*-values for tests for stationarity

Test	No drift, trend	Drift, no trend	Drift and trend
ADF	0.0100	0.0100	0.0100
PP	0.0100	0.0100	0.0100

The *p*-values of the ADF and PP test statistics are all less than 0.05 rejecting the null hypothesis of non-stationarity suggesting that the data is stationary at a 5% level of significance. Table 2 shows the descriptive statistics for the Covid-19 data.

**Table 2:** Descriptive statistics of the daily deaths for SA

Statistics	Value
Minimum	0.0000
Maximum	844.0000
Mean	157.2490
Std.dev	157.6464
Skewness	1.6926
Kurtosis	2.8619

**Fig. 2:**
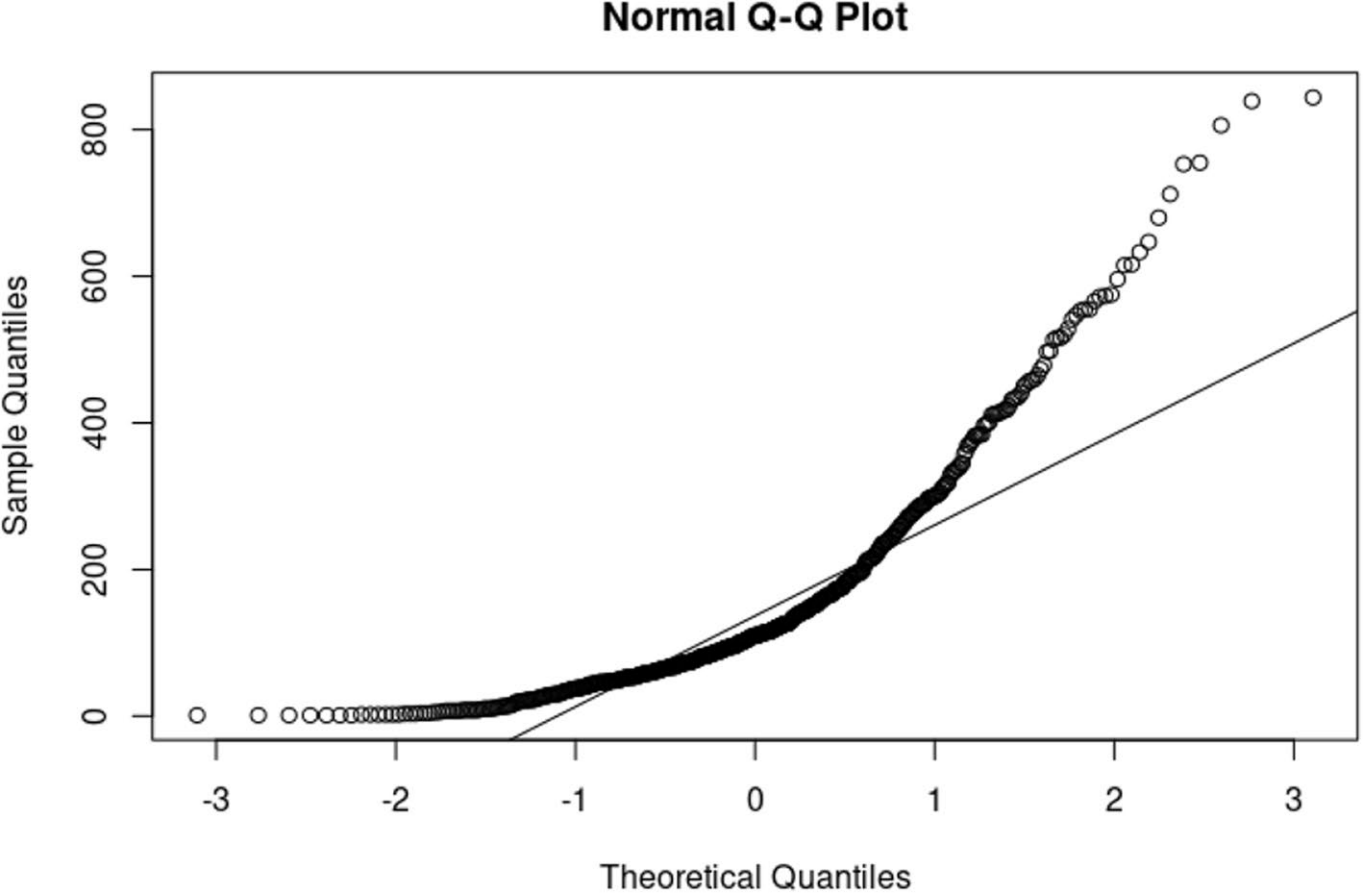
Q-Q plot for Covid 19 death data

In Table 2, the skewness and kurtosis show that the data is non-normal. Figure 2 shows the Q-Q plot for the data.

The Q-Q plot shows that the data points deviate from the line, indicating non-normality. In the tails, the data points move away from the line, which indicates possible heavy-tails. Table 3 shows the tests for normality.

**Table 3:** *P-*values for normality tests

Test	*P-*value
Shapiro–Wilk Test	< 0.0010
Jarque Bera Test	< 0.0010

In Table 3, the null hypothesis that the data points follow a normal distribution is rejected by the Shapiro–Wilk, and Jarque Bera tests a 5% significance level. The data is, therefore, non-normal.

**Fig. 3:**
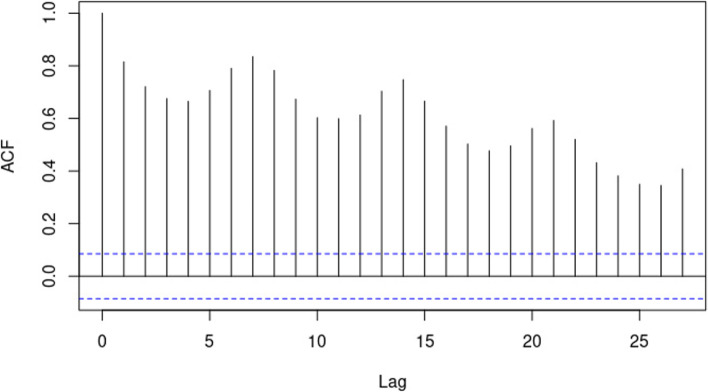
ACF plot for covid-19 daily deaths

To investigate the randomness of the data, Cox Stuart test result is provided in Table 4 and to further access the dependency of the daily deaths. Serial correlation is tested visually using an ACF plot in Fig. 3.

**Table 4:** *P-*values for tests for IID

Test	*P-*value
Cox Stuart Test	< 0.0010

Table 4 shows Cox Stuart test, which is the tests of randomness. The null hypothesis of randomness is rejected at 5% significance level, which means that the data points are not identically and independently distributed.

Figure 3 shows the ACF plot for the data.

In Fig. 3, it can be seen that the data possesses serial correlation. This is supported by Table 5, which are tests of serial correlation.

**Table 5:** *P-*values for tests for serial correlation and ARCH effects

Test	*P*-value
ARCH LM-test	< 0.0010
Durbin Watson test	< 0.0010

The Durbin Watson test reject the null hypothesis of no serial correlation. The ARCH LM-test rejects the null hypothesis of no ARCH effects in the data, meaning that the data has ARCH effects present. Table 6 shows the results for the test of structural breaks in the data.

**Table 6:** *P*-values for test for structural breaks

*Test*	*Trend*	*Intercept*	*Intercept and trend*
***Zivot-Andrews Unit Root test***	< 0.001	< 0.001	< 0.001
***Potential breakpoint***	431	316	319
	04–06-2021	09–02-2021	12–02-2021

In Table 6, the null hypothesis of no structural breaks is rejected at 5% significance level, which indicates that the data does exhibit structural breaks. The breaks are reported at 3 points/dates, 04 June 2020, 9 February 2021 and 12 February 2021.

**Fig. 4:**
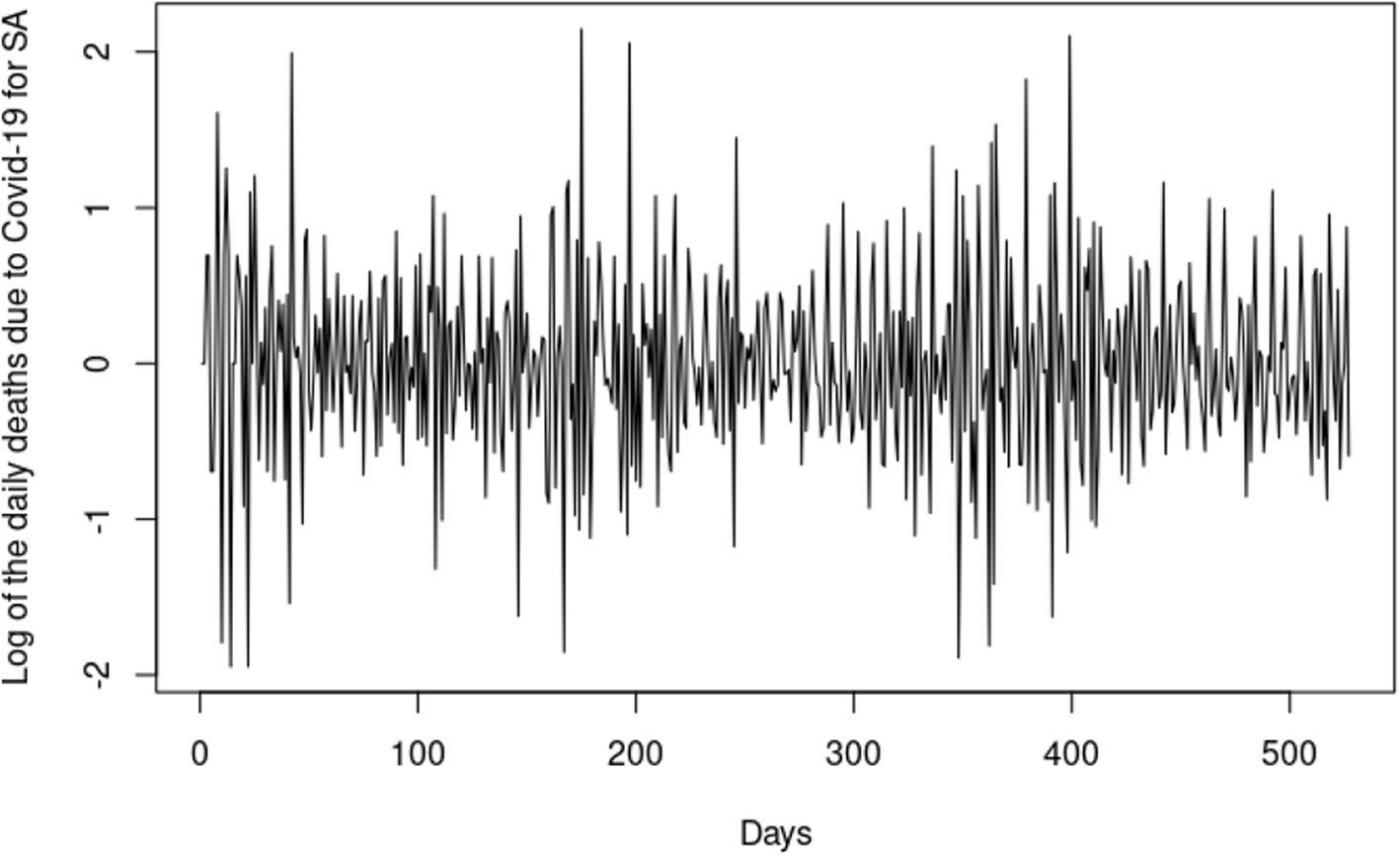
Time series plot of the log-returns of the daily deaths for Covid-19

To estimate the risk of death due to Covid-19, the concept of VaR is used. VaR estimation relies on certain distributional assumptions. Fitting a statistical distribution assumes that the data is stationary, independent, and identically distributed (iid.), with no serial correlation and no heteroscedasticity. The results previously reported show that the data is stationary, non-normal, not independently and identically distributed, exhibits serial correlation and heteroscedasticity. Thus, the data is transformed. Possible transformations are explored using the Box-Cox transformation technique, which revealed that the best transformation is the log transformation ($\lambda <0.5$). We define a log-return as ${r}_{t}=\mathrm{log}\left(\frac{{d}_{t}}{{d}_{t-1}}\right)$, where ${d}_{t}$ is the number of covid-19 related death at day *t* and ${d}_{t-1}$ is the number of covid-19 related death at day $t-1$. Figure 4 shows the plot for the log-returns of the daily deaths for Covid-19 for South Africa.

In Fig. 4, the transformed data seem to be stationary in the mean. However, there are still high and low fluctuations in the variance, which means volatility clustering is present in the data. Stationarity tests, namely the ADF and PP tests, are performed on the data to access the stationarity. Table 7 shows the *p*-values of the ADF and PP test statistic.

**Table 7:** *P*-values for tests for stationarity

Test	No drift, no trend	Drift, no trend	Drift and trend
ADF	< 0.0100	< 0.0100	< 0.0100
PP	< 0.0100	< 0.0100	< 0.0100

Table 7 shows the *p*-values of the stationarity tests, which are less than 0.05. The null hypothesis of non-stationarity is rejected at 5% significance level, and therefore the conclusion is that the log of the daily deaths is stationary. To explore more about the properties of the transformed data, the descriptive statistics were calculated. Table 8 shows the descriptive statistics for the transformed data.

**Table 8:** Descriptive statistics of the log returns of daily deaths for SA

Statistics	Value
Minimum	-18.6030
Maximum	18.6830
Mean	0.0115
Std.dev	2.7921
Skewness	0.2364
Kurtosis	35.9532

**Fig. 5:**
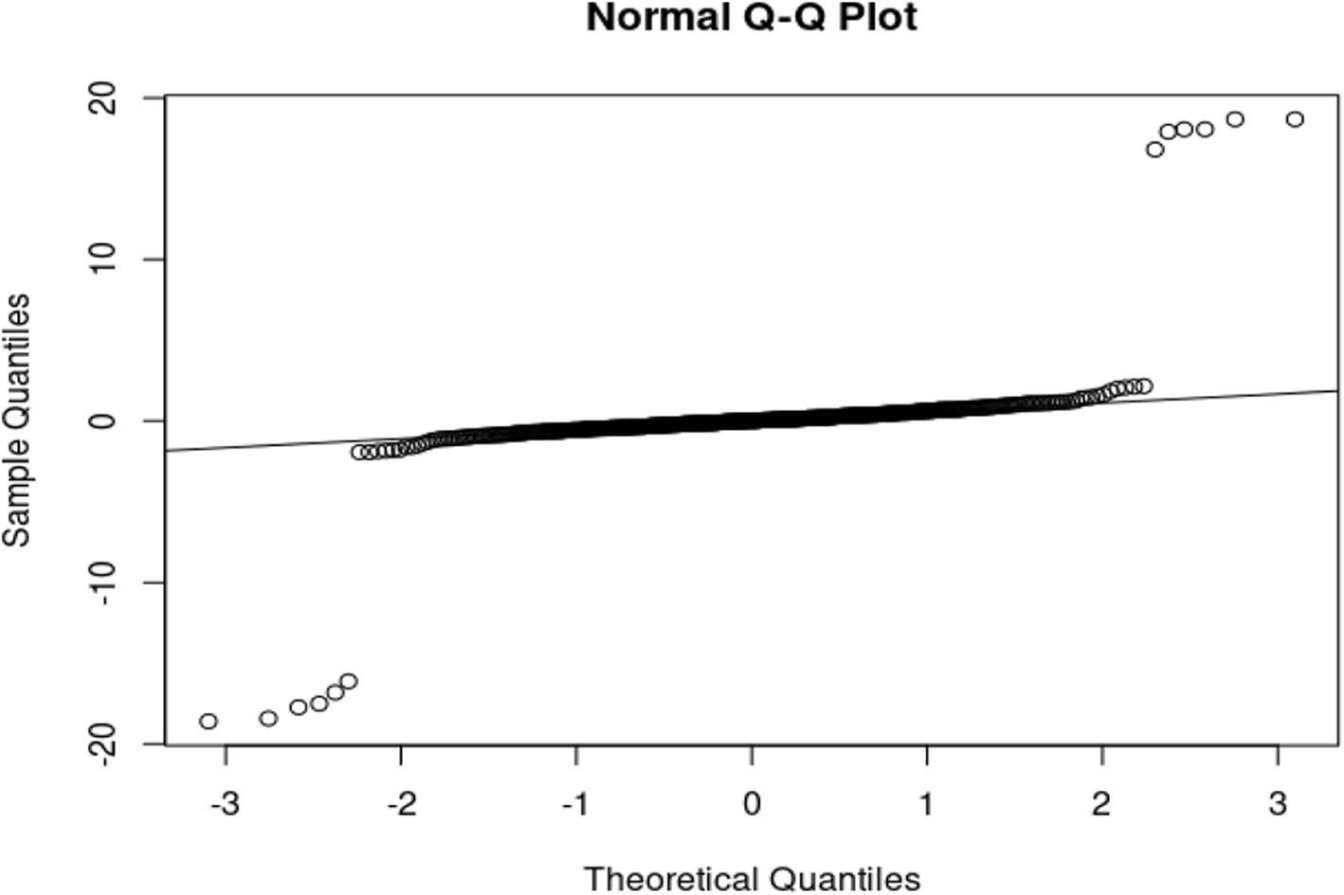
Q-Q plot for the daily deaths

The descriptive statistics in Table 8 reveal that the data is positively skewed, meaning that the tail on the right-hand side is longer than the tail on the left, this indicates the non-symmetric/non-normality of the data. This can further be supported by the excess kurtosis more significant than 0, indicating that the data has thick and heavier tails than the normal distribution (is leptokurtic). Figure 5 shows the Q-Q plot, which is a visual representation of a normality test. Table 9 are the normality test.

**Table 9:** *P-*values for normality tests

Test	*P-*value
Shapiro–Wilk Test	< 0.0010
Jarque Bera Test	< 0.0010

The Q-Q plot in Fig. 5 indicates that the data points move away from the line, more significantly on the right tail, which supports the results of the data being heavy-tailed. Furthermore, the Shapiro–Wilk test and the Jarque–Bera test in Table 9 have *p*-values less than 0.05, which is a clear indication that the null hypothesis of normality is rejected at 5% significance level. To investigate the randomness of the data, the Cox Stuart test is provided in Table 10, and to further assess if the data exhibits serial correlation and ARCH effects, Table 11 provides the ARCH LM test and Durbin Watson test.

**Table 10:** *P-*values for tests for IID

Test	*P-*value
Cox Stuart Test	0.7547

**Table 11:** *P-*values for tests for serial correlation and arch effects

Test	*P*-value
ARCH LM-test	< 0.0010
Durbin Watson test	> 0.9999

Table 10 shows that the data is identically and independently distributed. In Table 11, the null hypothesis of no serial correlation is not rejected at 5% level of significance by the Durbin Watson test. The Arch LM test rejects the null hypothesis of no ARCH effects, meaning that ARCH effects are present in the data.

### Summary of the EDA

From the exploratory data analysis, it can be concluded that the log-returns of the data exhibits,

- Stationarity
- Volatility clustering
- No-Serial correlation
- Arch effects (volatility clustering)
- i.i.d.
- three structural breaks
- Heavy tails (non-normal)

The summary of the exploratory data analysis leads to the following proposed model, a GARCH model to capture the volatility clustering and ARCH effects present in the data. Since the data revealed the presence of three structural breaks, a Markov Switching-GARCH-type model is further proposed to capture the regime changes in the data. Heavy-tailed distributions, namely, Student-*t* distribution (StD), Skewed Student-*t* distribution (SStD), normal reciprocal inverse Gaussian distribution (NRIG), and Pearson type IV distribution (PIVD) to capture the non-normality revealed by the data.

## Methods

### The GARCH model

The ARCH model by Eagle (1982) [16] is useful to forecast variance that changes over time. The GARCH model is a generalized version of the ARCH model, proposed by Bollerslev, (1986) [17]. The GARCH model allows for past conditional variances in the current conditional variance equation. The GARCH (*p*, *q*) model is written as,

1 $${h}_{t}=\omega +\sum\nolimits_{i=1}^{p}{\alpha }_{i}{a}_{t-i}^{2}+\sum\nolimits_{j=1}^{q}{\beta }_{j}{h}_{t-j}$$

From (1), the GARCH (1,1) is written as,

2 $${h}_{t}=\omega +{\alpha }_{1}{a}_{t-1}^{2}+{\beta }_{1}{h}_{t-1}$$

where ${h}_{t}$ is condional variance, ${a}_{t}={\sigma }_{t}{\epsilon }_{t},$ is the shock or random error, p and q are the number of lags in the model. and ${\epsilon }_{t}\sim N(\mathrm{0,1})$, the three positive numbers$\omega$, ${\alpha }_{1}$ and ${\beta }_{1}$ are the parameters of the GARCH $(\mathrm{1,1})$ process and ${\alpha }_{1}+{\beta }_{1}<1$. The forecast horizon, ${\widehat{h}}_{t+n}$ converges to

3 $${\widehat{h}}_{t+n}=\frac{\omega }{1-({\alpha }_{1}+{\beta }_{1})}$$

### The MS-GARCH model

The GARCH model has been useful for capturing conditional variance, but in the case of structural shifts, the GARCH model may bias upward the GARCH estimates if the shifts are not accounted for. This may lead to the misinterpretation of the estimates [18]. Hamilton (1989) [19] proposed a non-linear approach that accommodates changes in the regime. The approach uses a Markov switching regression to describe changes in the parameters of an autoregressive process.

#### Model specification

Ardia et al*.* (2018) [11] defined ${y}_{t}\in {\mathbb{R}}$ as the log-returns of an asset at time *t.* The mean of the log-returns is assumed to be zero and autocorrelated.

The Markov-switching can be specified as follows,

4 $${y}_{t}|\left({s}_{t}=k, {\mathfrak{T}}_{t-1}\right)\sim \mathcal{D}\left(0,{h}_{k,t},{\xi }_{k}\right)$$

where $\mathcal{D}\left(0,{h}_{k,t},{\xi }_{k}\right)$ is a continuous distribution with a mean zero, time changing variance ${h}_{k,t}$ and shape parameter ${\xi }_{k}$. ${s}_{t}$ is defined on the space $\left\{1,\dots ,K\right\}$ which follows a homogeneous Markov chain with the transition probability matrix $\mathbf{\rm P}\equiv {\left\{{p}_{i,j}\right\}}_{i,j=1}^{K}$, where.

${p}_{i,j}={\mathbb{P}}[{s}_{t}$=j|${s}_{t-1}=i]$. ${h}_{k,t}$ is the variance of ${y}_{t}$. Conditional on regime ${s}_{t}=k$, ${h}_{k,t}$ is defined as a function of past returns and the additional regime-dependant vector of parameters ${{\varvec{\theta}}}_{k}$.

The Markov-switching GARCH (1,1) is specified as,

5 $${h}_{k,t}\equiv {\omega }_{k}+{\alpha }_{k}{y}_{t-1}^{2}+{\beta }_{k}{h}_{k,t-1}$$

where $${\omega }_{k}>0,{ \alpha }_{k}>0, {\beta }_{k}\ge 0$$ and $${(\alpha }_{k}+{\beta }_{k})<1 (k=1,\dots ,K)$$, $${{\varvec{\theta}}}_{k}\equiv {({\omega }_{k}, { \alpha }_{k}, {\beta }_{k})}^{T}$$

#### Model estimation

The Markov-switching GARCH model makes use of the maximum likelihood (ML) or the Bayesian Markov chain Monte Carlo (MCMC) estimation techniques. Both these techniques require evaluating the likelihood function [20].

The specifications and methods of the model can be found in the **MSGARCH** package in R.

### Heavy-tailed distributions

#### Student-t distribution

The Gaussian GARCH model cannot explain the leptokurtosis present in asset returns [21]. it was suggested by Bollerslev (1987) [21] that the normality assumption of the error terms be replaced with that of the student-*t* distribution (S*t*D). The form of the distribution can be written as,

6 $$f(\mu_t{/Y}_{t-1}^p)=\frac{\Gamma\left[\frac12\left(\nu+1\right)\right]}{\pi^\frac12\Gamma\left[\frac12\nu\right]}{\lbrack\left(\nu-2\right)\sigma_t^2\rbrack}^{-\frac12}\left[1+\frac{\mu_t^2}{\left(\nu-2\right)\sigma_t^2}\right]^{-\frac12\left(\nu+1\right)}$$

The log-likelihood of the S*t*D then becomes:

7 $$\begin{array}{c}{L}_{St}\left(\theta \right)=\mathrm{ln}\left[\Gamma \left(\frac{v+1}{2}\right)\right]-\mathrm{ln}\left[\Gamma \left(\frac{v}{2}\right)\right]-\frac{1}{2}\mathrm{ln}\left[\pi \left(v-2\right)\right]\\ -\frac{1}{2}\sum_{t=1}^{T}\left[\mathrm{ln}{\sigma }_{t}^{2}+\left(1+v\right)\mathrm{ln}\left(1+\frac{\mu_{t}^{2}}{v-2}\right)\right]\end{array}$$

where $\nu$ (number of degrees of freedom), $\mu_{t}$ and ${\sigma }_{t}^{2}$ are the parameters.

#### Skewed student-t distribution

The S*t*D was extended by Fernandez and Steel (1998) [22] which introduced the addition of a skewness parameter. The density function can be expressed as:

8 $$f\left(\epsilon |\xi \right)=\frac{2}{\xi +\frac{1}{\xi }}\left[g\left(\frac{\epsilon }{\xi }\right) {I}_{\left[0,\infty \right)}\left(\epsilon \right)+g\left(\epsilon \xi \right){I}_{\left(-\infty ,0\right)}\left(\epsilon \right)\right]$$

$f\left(\epsilon |\xi \right)$ is a unimodal density and $\xi >0$ models the skewness.

Considering the random variable, ${z}_{t}=\frac{{\epsilon }_{t}-m}{s}$ with mean equal to zero and variance equal to one, the standardized skewed Student-*t* log-likelihood is:

9 $$\begin{array}{c}{L}_{SkSt}\left(\theta \right)=\mathrm{ln}\left[\Gamma \left(\frac{v+1}{2}\right)\right] -\mathrm{ln}\left[\Gamma \left(\frac{v}{2}\right)\right]-\frac{1}{2}\mathrm{ln}\left[\pi \left(v-2\right)\right]+\mathrm{ln}\left(\frac{2}{\xi +\frac{1}{\xi }}\right)\\ -\frac{1}{2}\sum_{t=1}^{T} \left[\mathrm{ln}{\sigma }_{t}^{2}+(1+v)\mathrm{ln}\left(1+\frac{s{z}_{t}+m}{v-2}{\xi }^{-{I}_{t}}\right)\right]\end{array}$$

where $${I}_{t}=\left\{\begin{array}{c}1~ if~ {z}_{t}\ge -\frac{m}{s}\\ -1 ~if~ {z}_{t}<-\frac{m}{s}\end{array}\right.$$

### Normal inverse Gaussian distribution

The normal inverse Gaussian (NIG) distribution is part of the generalised hyperbolic distribution (GHD). It was introduced by Barndorff-Nielsen (1994) [23]. To obtain the normal reciprocal inverse Gaussian (NRIG) distribution the parameter $\lambda$ is set to $\frac{1}{2}$.

The density function for a random variable $Y$ which follows an NRIG distribution can be defined as follows:

10 $${f}_{NRIG}\left(y|\mu ,\alpha ,\beta ,\sigma \right)=\frac{\sqrt{{\sigma }^{2}-{\beta }^{2}}{K}_{0}\left(\alpha \sqrt{{\sigma }^{2}+{\left(y-\mu \right)}^{2}}\right)}{\pi }\mathrm{exp}\left(\sigma \sqrt{{\sigma }^{2}-{\beta }^{2}}+\beta \left(y-\mu \right)\right)$$

### Pearson type IV distribution

The system of curves referred to as Pearsonian was initially developed by Karl Pearson [24]. This family of curves contain distributions like the PIVD, normal distribution, S*t*D and many more. The probability density function (pdf) of the PIVD is denoted by:

11 $$f\left(y\right)=c {\left[1+{\left(\frac{y-\lambda }{a}\right)}^{2}\right]}^{-n}\times \mathrm{exp}\left[-v{\mathit{tan}}^{-1} \left(\frac{y-\lambda }{a}\right)\right]$$

where $n>1/2$, $v$, $a$ and $\lambda$ are real-valued parameters, $-\infty <y<\infty$. The normalization constant $c$ depends on $n,v$ and $a$. The parameters $\lambda , v$ and $a$ are location, skewness, and scale parameters respectively, where $a>0$. The parameter $n$ is kurtosis.

The log-likelihood of the PIVD can be expressed as follows:

12 $${L}_{PIVD}\left(\theta \right)=N\mathrm{ln}k\pm m{\sum }_{i=1}^{N}\mathrm{ln}\left[1+{\left(\frac{y-\lambda }{a}\right)}^{2}\right]-{\sum }_{i=1}^{N}{\mathrm{tan}}^{-1}\left(\frac{{y}_{i}-\lambda }{a}\right)$$

$N$ represents the number of observed data points in ${x}_{i}$. For further explanations, see [25].

### Value-at-risk

Value-at-risk (VaR) can be defined as the maximum loss, that with a stated probability should not be exceeded during a given period. In market risk management, VaR measures the maximum plausible loss of a portfolio of a financial instrument over a given period [13].

For a stated probability, the VaR is defined as the *p*th quantile of $F$ such that,

13 $${VaR}_{p}={F}^{-1}(1-p)$$

${F}^{-1}$ is the quantile function.

To access the precision of the results, backtesting is done on the VaR estimates. The Kupiec likelihood ratio test by Kupiec (1995) [26] is utilised. The Kupiec test is based on that a proper model should have a fraction of violations of VaR estimates near the respective tail probability $\alpha$ [27].

The null hypothesis of the Kupiec likelihood ratio test is that the expected proportions of violations is equal to $\alpha$. The test statistic can be written as:

14 $${LR}_{UC}=-2\mathrm{ln}\left(\frac{{\left(\frac{{y}^{\alpha }}{N}\right)}^{{x}^{\alpha }}{\left(1-\frac{{y}^{\alpha }}{N}\right)}^{N-{x}^{\alpha }} }{\left({\alpha }^{{x}^{\alpha }}{\left(1-\alpha \right)}^{N-{x}^{\alpha }}\right)}\right)$$

${y}^{\alpha }$ counts the amount of times where observed returns fall beneath (for long positions) or above (for short positions) the VaR estimate at level $\alpha$.

The return process for the portfolio is defined as,

15 $${r}_{t}=\mu +{z}_{t}\sqrt{{h}_{t}}$$

where ${h}_{t}$ is the volatility process and ${z}_{t}$ are the standardized residuals which are written as,

16 $${z}_{t}=\frac{{r}_{t}-\mu }{\sqrt{{h}_{t}}}$$

The VaR estimates forecast for the next day is defined as,

17 $${VaR}_{t+1}=current~ position\times \left(\mu -{z}_{q}\sqrt{{\widehat{h}}_{t+1}}\right)$$

## Empirical results

In this section, the data is described, and the data source is provided. Furthermore, the exploration of the data is done to reveal the intrinsic properties of the data. The suggested model-MS-GARCH model is fitted with heavy-tailed distributions, and the model adequacy results are described.

A GARCH (1,1) model with normal distribution governing the innovations was fitted to the transformed data. Table 12 shows the results of the fitted GARCH (1,1) model with normal distribution governing the residuals.

**Table 12:** ML Parameter estimates of the GARCH (1,1) model

Parameter	Estimate	Standard error	*P*-value
$\widehat{\omega }$	0.0112	0.0028	< 0.0001
$\widehat{\alpha }$	0.1060	0.0212	< 0.0010
$\widehat{\beta }$	0.0870	0.0119	< 0.0001

In Table 12, all the parameter estimates for the GARCH (1,1) model are statistically significant at 5% level of significance. This means that a GARCH (1,1) model would be appropriate to fit the data. To check for asymmetric effects, the sign-bias test was used. Table 13 shows the results of the sign bias test.

**Table 13:** Sign and size test

Test	*T-value*	*P-*value
Sign Bias	1.1973	0.2317
Negative Sign Bias	2.3370	< 0.0198
Positive Sign Bias	0.2160	0.8291
Joint Effect	6.1130	0.1062

The sign bias test has a *p*-value greater than 0.05, therefore the null hypothesis of no asymmetric effects is not rejected at 5% level of significance. This means that an appropriate model should be symmetric. The next section of this paper explores a GARCH-type model with no asymmetric effects.

### Markov-switching GARCH model

In this section, an MS(k)-GARCH(1,1) model is fitted, the extracted residuals are tested for normality, and the appropriate heavy-tailed distributions are fitted to the standard residuals. The goodness of fit is also provided for the fitted models.

Due to the presence of three structural breaks in the data and the GARCH(1,1) best fitting the data, symmetric MS(*k*)-GARCH(1,1) model with three structural breaks ($k=3$) is fitted to the data. Table 14 shows the results.

**Table 14:** ML parameter estimates of the MS(3)-GARCH (1,1) model

Parameter	Estimate	Standard error	*P*-value
${\widehat{\alpha }}_{01}$	0.0666	< 0.0001	< 0.0001
${\widehat{\alpha }}_{11}$	0.0033	< 0.0001	< 0.0001
${\widehat{\beta }}_{1}$	0.0001	< 0.0001	< 0.0001
${\widehat{\alpha }}_{02}$	0.0166	< 0.0001	< 0.0001
${\widehat{\alpha }}_{12}$	0.1115	< 0.0001	< 0.0001
${\widehat{\beta }}_{2}$	0.8884	< 0.0001	< 0.0001
${\widehat{\alpha }}_{03}$	0.1909	< 0.0001	< 0.0001
${\widehat{\alpha }}_{13}$	0.9851	< 0.0001	< 0.0001
${\widehat{\beta }}_{3}$	0.0142	< 0.0001	< 0.0001
${\widehat{\rho }}_{1}$	0.1442	< 0.0001	< 0.0001
${\widehat{\rho }}_{2}$	0.1507	< 0.0001	< 0.0001

Table 14 shows the parameter estimates, standard error, and *p*-values of the parameters of the MS-GARCH model. The *p*-values are less than 0.05, which means that the parameters are all statistically significant at a 5% significance level.

After extracting the residuals, they are tested for normality. Table 15 shows the basic statistics of the standardized residuals.

**Table 15:** Descriptive statistics for the residuals

Statistics	Value
Minimum	-5.7477
Maximum	6.9690
Mean	0.0385
Std.dev	1.1741
Skewness	0.5174
Kurtosis	6.2371

**Fig. 6:**
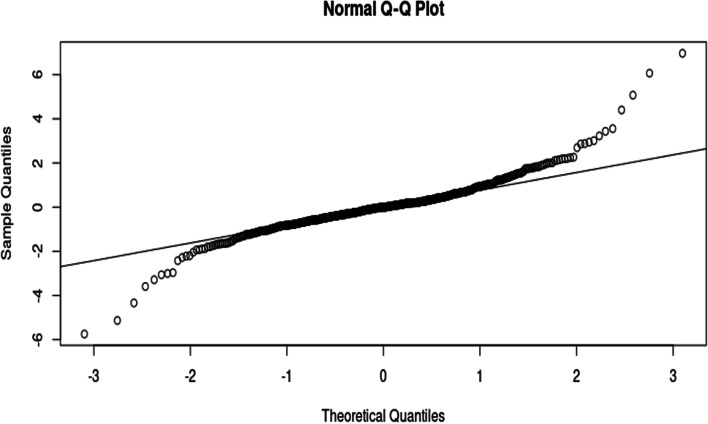
Q-Q plot for the residuals

Table 15 shows that the residuals are positively skewed and that the residuals are extremely leptokurtic, which is seen by the big excess kurtosis. All this indicates that the residuals are heavy-tailed. Figure 6 shows the Q-Q plot for the residuals.

The Q-Q plot in Fig. 6 shows the data points for the residuals deviating from the normal line, especially on the right-hand side. This supports the results of the kurtosis and skewness that the data exhibits heavier tails than the normal distributions. Table 16 shows the normality tests for the residuals.

**Table 16:** *P-*values for normality tests for the residuals

Test	*P-*value
Shapiro–Wilk Test	< 0.0010
Jarque Bera Test	< 0.0010

The Shapiro–Wilk and Jarque Bera tests in Table 16 have *p*-values less than 0.05, which means that the residuals do not follow a normal distribution. Since the residuals are non-normal and heavy-tailed, we capture the non-normality by fitting heavy-tailed distributions to the residuals. The Anderson–Darling (AD) statistic is used to check whether the fitted distributions fit the data well. Table 17 shows the parameter estimates for the MS(3)-GARCH(1,1)-with the heavy-tailed distributions governing the innovations and the *p*-value for the Anderson Darling (AD) statistic.

**Table 17:** ML Parameter estimates for MS(3)-GARCH(1,1)-model with heavy-tailed innovations

Model	Parameter	Estimate	*P*-value for the AD statistic
***MS-GARCH-S*t*D***	${\widehat{\mu }}_{s\mathrm{td}}$ ${\widehat{\sigma }}_{s\mathrm{td}}$ ${\widehat{\nu }}_{s\mathrm{td}}$	-0.0187 1.3348 2.7672	0.6193
***MS-GARCH-SS*t*D***	${\widehat{\mu }}_{ss\mathrm{td}}$ ${\widehat{\sigma }}_{ss\mathrm{td}}$ ${\widehat{\nu }}_{ss\mathrm{td}}$ $\widehat{\xi }$	0.0396 1.3209 2.8148 1.1032	0.9593
***MS-GARCH-NRIGD***	${\widehat{\lambda }}_{NRIGD}$ ${\widehat{\alpha }}_{NRIGD}$ $\widehat{\delta }$ ${\widehat{\beta }}_{NRIGD}$ ${\widehat{\mu }}_{NRIGD}$	0.5 1.0369 0.3419 0.0924 -0.0787	0.9636
***MS-GARCH-PIVD***	$\widehat{m}$ ${\widehat{\nu }}_{PIVD}$ ${\widehat{\lambda }}_{PIVD}$ ${\widehat{\alpha }}_{PIVD}$	2.3455 0.3184 1.3856 1.1699	0.9812

From Table 17, the *p*-values of the AD statistic are greater than 0.05 indicating that the fitted MS(3)-GARCH(1,1)-models with heavy-tailed distributions fit the data well. Thus, we estimate the VaR values at different VaR levels and backtest the VaR values using the Kupeic likelihood ratio test.

### VaR estimation and backtesting

In Finance, VaR measures the risk of loss for investments. It estimates by how much a set of investments might lose (with a certain probability), given normal market conditions, in a given period such as a day. This paper looks at how many people will die each day at a given/specified probability level in South Africa. Table 18 shows the VaR estimates for the MS(3)-GARCH(1,1) model fitted with heavy-tailed distributions.

**Table 18:** VaR estimates for MS(3)-GARCH(1,1) model with heavy-tailed distributions

VaR Levels				
Model	***90%***	***95%***	***97.5%***	***99%***
*MS-GARCH-*S*t*D	1.1590	1.6936	2.3283	3.3976
*MS-GARCH -*SS*t*D	1.2484	1.8413	2.5456	3.7292
*MS-GARCH -*NRIGD	1.3240	1.9420	2.5790	3.4418
*MS-GARCH -PIVD*	1.2500	1.8610	2.5967	3.8466

From Table 18, we can observe that the MS(3)-GARCH(1,1) model combined with NRIGD and PIVD produce VaR estimates that are closer to those of the empirical distribution at most VaR levels. This is not surprising, as we have seen that the MS(3)-GARCH(1,1)-NRIGD and MS(3)-GARCH(1,1)-PIVD provide a better fit to the data than MS(3)-GARCH(1,1)-S*t*D and MS(3)-GARCH(1,1)-SS*t*D. In order to check the adequacy of these VaR estimates, the Kupiec likelihood ratio test is used. The *p*-values of the Kupiec likelihood ratio test at various significance levels are shown in Table 19.

**Table 19:** *P*-values for the Kupiec test for backtesting the VaR estimates using MS(3)-GARCH (1,1) model with heavy-tailed distributions

VaR Levels				
Model	***90%***	***95%***	***97.5%***	***99%***
*MS-GARCH-*S*t*D	0.0952	0.0305	**0.8136**	0.7070
*MS-GARCH-*SS*t*D	0.4948	**0.7862**	0.8135	0.6020
*MS-GARCH-*NRIGD	**0.9181**	0.7356	**0.8136**	**0.9538**
*MS-GARCH-*PIVD	0.4948	**0.7862**	**0.8136**	0.6020

Backtesting confirms which model is the best for estimating risk, this is the model with the highest *p*-value. Table 19 shows that the MS(3)-GARCH(1,1)-NRIGD is the best model at 90%. At 95%, the MS(3)-GARCH(1,1)-SS*t*D and MS(3)-GARCH(1,1)-PIVD are the best models, and MS(3)-GARCH(1,1)-S*t*D, MS(3)-GARCH(1,1)-NRIGD, and MS(3)-GARCH(1,1)-PIVD are the best models at 97.5%. The MS(3)-GARCH(1,1)-NRIG is the best model at 99%. Thus, using the Kupiec likelihood ratio test for model selection, at 95% level, the best fitting models are the MS(3)-GARCH(1,1)-SS*t*D and MS(3)-GARCH(1,1)-PIVD.

We use MS (3)-GARCH (1,1) combined with heavy tailed distributions, (3) and (17) to forecast the out-of-sample minimum number of daily deaths i.e. minimum deaths due to Covid-19 for 10 days head. In (17), to predict the minimum number of deaths at time *t,* we consider, ${\mu }_{T=1}$ the average number of deaths due to Covid-19 for the period 27 March 2020 to 26 August 2021, which is the in-sample data, ${\mu }_{T=2}$ is the average number of deaths for the period 28 March 2020 to 27 August 2021, Thus, includes the forecasted number of deaths on 27 August 2021. ${\mu }_{T}$ for the periods $T=3 \mathrm{up to} 10$ are estimated in the same way. In Table 20, ${\mathbf{V}}_{\mathbf{T}}$ denotes the actual/observed deaths per day, and ${\mathbf{V}}_{\mathbf{T}+1}$ denotes the forecasted death value. To assess the accuracy of the models in predicting the number of daily deaths due to Covid-19 infection with a 95% probability, we present the actual observed and predicted values in the forecasting period in Table 20.

**Table 20:** Out-of-Sample forecast of daily number of deaths for the fitted models 95% VaR levels

Day	${\varvec{O}}{\varvec{b}}{\varvec{s}}{\varvec{e}}{\varvec{r}}{\varvec{v}}{\varvec{e}}{\varvec{d}}\boldsymbol{ }{\varvec{d}}{\varvec{e}}{\varvec{a}}{\varvec{t}}{\varvec{h}}{\varvec{s}}$	${\varvec{P}}{\varvec{I}}{\varvec{V}}{\varvec{D}}$	***StD***	***SStD***	***NRIG***
$1$	361	322	182	328	308
*2*	274	290	104	282	252
*3*	134	247	84	250	254
*4*	235	218	70	209	170
*5*	431	304	62	299	211
*6*	235	275	51	281	182
*7*	418	524	47	503	200
*8*	247	317	34	322	162
*9*	182	114	23	118	57
*10*	76	41	15	50	34
***RMSE***		**74**	**197**	**77**	**117**

In Table 20, it can be said that with a probability of 95%, the number of deaths that will occur at 1 day ahead (27^th^ August 2021) will be greater than $322.$ We illustrate how we forecast ${\mathbf{V}}_{\mathbf{T}+1}$ using (3) and (19): ${\widehat{h}}_{t+1}=\frac{0.0666}{1-(0.0033+0.0001)}$ and ${VaR}_{t+1}=357\times \left(\left|1.3856-1.8610\sqrt{{\widehat{h}}_{t+1}}\right|\right)$, for MS(3)-GARCH(1,1)-PIVD hybrid model. We assess the predictive accuracy of the fitted models at 95% using the Root Mean Square Error, defined as

18 $$RMSE=\sqrt{\frac{1}{n}\sum\nolimits_{T=1}^{n}({{V}_{T+1}-{V}_{T})}^{2}}$$

The best fitting model has a RMSE value of 74. The suggest that the proposed model is reasonable and can be used as an early warning tool to inform the government health officials and other stakeholders about the future daily number of deaths for South Africa.

In view of the existing challenges in the provision of healthcare in South Africa, reliable and accurate short-term forecasts of Covid-19 related deaths are critical to ensure optimal allocation of scarce resources. Having accurate and reliable information on the possible number of deaths in advance is a fundamental strategy to prepare for the number of beds required for hospitalisation, ventilators and health practitioners needed to mitigate against the predicted number of deaths due to Covid-19 infection on forecasted days.Thereby, reducing the effect of untimely deaths due to Covid-19 infection. The importance of predicting Covid-19-related deaths measures the effectiveness of all the mitigating efforts by Governments, such as vaccinations and lockdowns. It also measures the extent of the damage caused by Covid-19 infection.

## Conclusion

The SARS-CoV-2 pandemic is a global issue, threatening the human population and the economy.The estimation of deaths due to Covid-19 may interest health analysts, policymakers, biostatisticians. This study estimates the risk of death due to the Covid-19 virus using a Markov-Switching GARCH type model combined with heavy-tailed distributions. The risk of death is estimated using the concept of VaR as it is used in financial time series. The results show that the MS(3)-GARCH(1,1)–NRIGD is the best model at 90%. At 95% the MS(3)-GARCH(1,1)–SStD and MS(3)-GARCH(1,1)-PIVD are the best models, and MS(3)-GARCH(1,1)–StD, MS(3)-GARCH(1,1)–NRIGD, MS(3)-GARCH(1,1)–PIVD are the best at 97.5%. The MS(3)-GARCH(1,1)–NRIG is the best model at 99%. Overall MS(3)-GARCH(1,1)-NRIGD is the best model, it outperforms all the models at almost all the levels. The VaR results showed that with a probability of 95%, the minimum number of daily deaths that will occur for the next 10 days is reasonable with a root mean square error of 74. This suggests that the model has a high predictive accuracy and can be used in making informed decisions by government health officials and policy-makers.

## Data Availability

The datasets generated and/or analysed during the current study are available in the University of Pretoria repository https://github.com/dsfsi/covid19za/blob/master/data/.
